# Acute Diffusion Tensor and Kurtosis Imaging and Outcome following Mild Traumatic Brain Injury

**DOI:** 10.1089/neu.2021.0074

**Published:** 2021-08-23

**Authors:** Jonas Stenberg, Live Eikenes, Kent Gøran Moen, Anne Vik, Asta K. Håberg, Toril Skandsen

**Affiliations:** ^1^ Department of Neuromedicine and Movement Science, Norwegian University of Science and Technology (NTNU), Trondheim, Norway.; ^2^ Department of Neurosurgery, St Olavs Hospital, Trondheim University Hospital, Trondheim, Norway.; ^3^ Department of Circulation and Medical Imaging, Norwegian University of Science and Technology (NTNU), Trondheim, Norway.; ^4^ Department of Radiology, Nord-Trøndelag Hospital Trust, Levanger Hospital, Levanger, Norway.; ^5^ Department of Radiology and Nuclear Medicine, St Olavs Hospital, Trondheim University Hospital, Trondheim, Norway.; ^6^ Department of Physical Medicine and Rehabilitation, St Olavs Hospital, Trondheim University Hospital, Trondheim, Norway.

**Keywords:** biomarkers, brain concussion, diffusion kurtosis imaging, diffusion tensor imaging, post-concussion syndrome

## Abstract

In this prospective cohort study, we investigated associations between acute diffusion tensor imaging (DTI) and diffusion kurtosis imaging (DKI) metrics and persistent post-concussion symptoms (PPCS) 3 months after mild traumatic brain injury (mTBI). Adult patients with mTBI (*n* = 176) and community controls (*n* = 78) underwent 3 Tesla magnetic resonance imaging (MRI) within 72 h post-injury, estimation of cognitive reserve at 2 weeks, and PPCS assessment at 3 months. Eight DTI and DKI metrics were examined with Tract-Based Spatial Statistics. Analyses were performed in the total sample in uncomplicated mTBI only (i.e., without lesions on clinical MRI), and with cognitive reserve both controlled for and not. Patients with PPCS (*n* = 35) had lower fractional anisotropy (in 2.7% of all voxels) and kurtosis fractional anisotropy (in 6.9% of all voxels), and higher radial diffusivity (in 0.3% of all voxels), than patients without PPCS (*n* = 141). In uncomplicated mTBI, only fractional anisotropy was significantly lower in patients with PPCS. Compared with controls, patients with PPCS had widespread deviations in all diffusion metrics. When including cognitive reserve as a covariate, no significant differences in diffusion metrics between patients with and without PPCS were present, but patients with PPCS still had significantly higher mean, radial, and axial diffusivity than controls. In conclusion, patients who developed PPCS had poorer white matter microstructural integrity acutely after the injury, compared with patients who recovered and healthy controls. Differences became less pronounced when cognitive reserve was controlled for, suggesting that pre-existing individual differences in axonal integrity accounted for some of the observed differences.

## Introduction

After a mild traumatic brain injury (mTBI), a minority of patients experience disabling symptoms for several months to years, referred to as persistent post-concussion symptoms (PPCS).^1^ The evolution of PPCS is likely determined both by individual, pre-existing vulnerability to health problems, and by the injury itself,^1^ yet the neural mechanisms behind PPCS are poorly understood. In particular, the role of trauma-induced axonal injury is debated. Few patients have visible traumatic lesions on computed tomography or clinical magnetic resonance imaging (MRI; i.e., complicated mTBI), and studies of the association between such macrostructural lesions and outcome have often been inconclusive.^2^ However, most mTBI pathology is likely expressed on the axonal and microstructural level, which has led to an enormous research interest in diffusion MRI,^3,4^ and microstructural abnormalities in white matter after mTBI have been revealed with diffusion tensor imaging (DTI).^3^ Yet, the significance of such abnormalities on outcome is unclear for a number of reasons.

First, in most previous studies on PPCS, DTI has been performed in the subacute or chronic phase after the mTBI, often concurrently with PPCS assessment, and mostly in small samples. Differences in DTI metrics between patients with and without PPCS have been demonstrated in some, but not all, of these subacute or chronic phase studies.^5–15^ From a clinical perspective, however, it would be useful to identify patients at risk of PPCS early, and acute DTI could potentially serve as a biomarker for poor long-term outcome. So far, there is a paucity of studies examining whether acute (i.e., within the first few days after injury) DTI predicts later PPCS.

Second, most mixed-mechanism mTBI study samples comprise a certain proportion of patients with complicated mTBI,^8,14^ and the observed differences in diffusion measures between patients and controls could primarily be driven by a more pronounced pathology in the brain tissue of patients with complicated mTBI. Thus, the added value of DTI to clinical MRI, in PPCS prediction remains to be established.

Third, abnormal diffusion is not specific for brain trauma.^16–18^ Accordingly, if diffusion measures are different in patients with PPCS, it is of interest whether these deviations were induced by the head trauma, were pre-existing, or both. Consequently, pre-existing characteristics previously shown to be associated both with PPCS and diffusion measures need to be controlled for. A potential candidate is cognitive reserve, estimated by general mental ability or intelligence.^19–25^

Finally, diffusion kurtosis imaging (DKI) has been proposed to be more sensitive than DTI. DKI does not assume a Gaussian distribution of diffusion and may be superior in identifying microstructural abnormalities in areas with high heterogeneity.^26,27^ Deviating DKI metrics have been reported in both white and gray matter in the acute to chronic phase following mTBI,^28–35^ but the association between DKI metrics and PPCS is unclear.^15,32,34,35^

To fill these knowledge gaps, the current study includes a large representative sample of patients with mixed-mechanism mTBI who underwent both DTI and DKI in the acute phase.^36,37^ The aim was to compare diffusion metrics between patients who later developed PPCS, patients without PPCS, and healthy controls in the total sample, in patients with uncomplicated mTBI, and with cognitive reserve both controlled for and not.

## Methods

### Participants

The patients with mTBI were part of the population-based Trondheim mTBI follow-up study (total *n* = 378), recruited from April 2014 to December 2015 at two emergency departments: a level 1 trauma center in Trondheim, Norway, and at the Trondheim Municipal Emergency clinic, a general practitioner-run, outpatient clinic.^36^ A total of 199 patients participated in an extended follow-up study including acute MRI. Inclusion criteria were age 16 to 59 years and having sustained a TBI,^38^ defined as mild per the World Health Organization Collaborating Center Task Force on Mild Traumatic Brain Injury criteria (detailed inclusion and exclusion criteria are reported in the Supplementary Material).^39^ Age-, sex-, and education-matched community controls (*n* = 78) were recruited among hospital and university staff, students, and acquaintances of staff, students, and patients. The control group was found to be comparable to the mTBI group on a broad range of personal factors.^40^ The study was approved by the regional committee for research ethics (REK 2013/754), and participants and parents of participants younger than 18 years gave informed consent.

### Magnetic resonance imaging

Patients with mTBI underwent MRI on a 3T Siemens Skyra system (Siemens Healthcare, Erlangen, Germany) with a 32-channel head coil, the majority (91%) within 72 h after injury (mean 52 h ± 19 h, range 5-130 h). A radiologist and a resident in radiology reported the following MRI sequences: 1) three-dimensional (3D) T1-weighted magnetization-prepared rapid acquisition with gradient echo (MPRAGE); 2) two-dimensional diffusion-weighted imaging (DWI); 3) 3D T2 space; 4) 3D T2-weighted fluid-attenuated inversion recovery; and 5) 3D T2-weighted susceptibility-weighted imaging, previously reported in detail.^37^ Patients with visible traumatic intracranial lesions had complicated mTBI, while those without had uncomplicated mTBI.

The DTI/DKI sequence was a single-shot balanced-echo EPI sequence acquired in 30 non-collinear directions with 3 b-values (b = 0, b = 1000 and, b = 2,000 sec/mm^2^). The following parameters were used: repetition time 8800 msec, echo time 95 msec, field of view 240 × 240 mm, slice thickness 2.5 mm, acquisition matrix 96 × 96. Sixty transversal slices with no gaps were acquired, giving full brain coverage. Five images without diffusion weighting were acquired to increase signal-to-noise ratio. To correct for image distortion, two additional b0 images were acquired with opposite phase encoding polarity.^41^ Details on DTI and DKI data processing are described in the Supplementary Material.

### Persistent post-concussion symptoms

PPCS were assessed at 3 months after injury with the British Columbia Postconcussion Symptom Inventory (BC-PSI).^42^ BC-PSI consists of 13 core symptoms distributed over four symptom categories (i.e., somatic, emotional, cognitive, and sleep disturbance), and three life problems, distributed over two additional symptom categories (i.e., reduced tolerance to alcohol; preoccupation with the symptoms and fear of permanent brain damage). The respondents rate the frequency and severity of each symptom during the past 2 weeks. These scores are combined into a single score representing the frequency and severity of each symptom (range 0-4). The sum of the 13 core symptoms constitutes the total score. PPCS was defined as having ≥3 core symptoms rated at least moderate (score ≥3), or a total score of 13–52 (i.e., 52 is the highest possible score).^40^

### Estimated pre-injury intelligence and cognitive reserve

The Vocabulary subtest from the Wechsler Abbreviated Scale of Intelligence,^43,44^ was used as an estimate of pre-injury intelligence and a proxy of cognitive reserve^45^ and was administered 2 weeks after the injury. The task entails explaining the meaning of 42 words; 2 points are given for a correctly explained word and 1 point for a partly correct explanation. The Vocabulary subtest is considered an estimate of general mental ability^46^ and test performance has been shown to be unaffected by cognitive impairment following mTBI.^47,48^

### Statistical analysis

Demographic variables were examined with t-tests, Mann-Whitney U-tests, and chi-squared tests. Voxel-wise statistical analysis of the diffusion data was performed using Tract-Based Spatial Statistics (TBSS).^49^ Differences in diffusion metrics between patients with PPCS, patients without PPCS, and the control group (i.e., three comparisons) were analyzed with the Randomize tool in fMRIB Software Library, a non-parametric, permutation-based method using threshold-free cluster enhancement with correction for multiple comparisons (family-wise error rate).^50^ A *p* value of <0.05, corrected for multiple comparisons, was considered statistically significant. Age, age^2^, sex, and scanner upgrade (due to scanner upgrade from version D13 to E11 during the inclusion period) were controlled for in all analyses. Because diffusion metrics show a non-linear association with age through adulthood (e.g., FA peaks around the age of 30), both age and age^2^ were included in all models.^51^ The lowest corrected *p* value and the number of significant voxels in each contrast were extracted. Eight diffusion metrics were examined: fractional anisotropy (FA), mean diffusivity (MD), axial diffusivity (AD), radial diffusivity (RD), kurtosis fractional anisotropy (KFA), mean kurtosis (Kmean), axial kurtosis (Kax), and radial kurtosis (Krad).

All analyses were performed with the patients with complicated mTBI both included (the total sample) and excluded (the uncomplicated sample). Analyses also were performed with vocabulary scores included as an additional covariate, to investigate whether differences in cognitive reserve between groups affected the results.

## Results

### Participant characteristics

A total of 186 out of the 199 patients in the extended follow-up had DTI and DKI data that passed quality control, and 176 of these were assessed for PPCS at 3 months, constituting the study sample (88% of the patients in the extended follow-up). Eighteen (10%) had complicated mTBI (Table 1). There were no significant differences between the included patients with mTBI and controls regarding age, sex, years of education, or vocabulary scores (Table 1). Differences between included and excluded participants are presented in Table 1 of the Supplementary Material.

**Table 1.** Participant Characteristics and Injury-Related Factors

	mTBI group	Control group	*p*^1^	PPCS+	PPCS-	*p*^2^
*n*	176	78		35	141	
Age, years, median (IQR)	28.1 (22.0)	27.6 (20.0)	0.995^3^	31.5 (25.3)	27.0 (21.7)	0.411^3^
Female sex, *n* (%)	65 (36.9)	30 (38.5)	0.816^4^	19 (54.3)	46 (32.6)	0.017^4^
Education, years, median (IQR)	13.0 (4.0)	13.0 (4.0)	0.663^3^	12.0 (4.0)	13.0 (4.0)	0.135^3^
Vocabulary						
T score, mean (SD)	50.9 (9.2)	51.3 (8.1)	0.764^5^	47.5 (9.1)	51.6 (9.1)	0.042^5^
Raw score, mean (SD)	57.4 (8.6)	57.7 (7.6)	0.704^6^	54.6 (9.0)	58.0 (8.4)	0.047^6^
Cause of injury, *n* (%)						
Fall	68 (38.6)			15 (42.9)	53 (37.6)	
Bicycle	33 (18.8)			5 (14.3)	28 (19.9)	
Violence	23 (13.1)			9 (25.7)	14 (9.9)	
Sports accidents	21 (11.9)			0 (0)	21 (14.9)	
Motor vehicle accidents	17 (9.7)			5 (14.3)	12 (8.5)	
Hit by object	12 (6.8)			0 (0)	12 (8.5)	
Other	1 (0.6)			1 (2.9)	0 (0)	
Unknown	1 (0.6)			0 (0)	1 (0.7)	
GCS score, *n* (%)						
13	4 (2.3)			0 (0)	4 (2.8)	
14	25 (14.2)			7 (20.0)	18 (12.8)	
15	136 (77.3)			25 (71.4)	111 (78.7)	
unknown	11 (6.3)			3 (8.5)	8 (5.7)	
LOC, *n* (%)						0.103^4^
Yes	85 (48.3)			15 (42.9)	70 (49.6)	
No	30 (17.0)			3 (8.6)	27 (19.1)	
unknown/not witnessed	61 (34.7)			17 (48.6)	44 (31.2)	
PTA, *n* (%)						0.002^4^
< 1 h	123 (69.9)			17 (48.6)	106 (75.2)	
1–24 h	53 (30.1)			18 (51.4)	35 (24.8)	
Complicated mTBI, *n* (%)						0.006^4^
Yes	18 (10.2)			8 (22.9)	10 (7.1)	
No	158 (89.8)			27 (77.1)	131 (92.9)	
Level of care, *n* (%)						
Not admitted	124 (70.5)			20 (57.1)	104 (73.8)	
Observed <24 h	27 (15.3)			6 (17.1)	21 (14.9)	
Admitted neurosurgery department	16 (9.1)			7 (20.0)	9 (6.4)	
Admitted other department	9 (5.1)			2 (5.7)	7 (5.0)	

19 patients with mTBI, and 4 controls did not perform the Vocabulary subtest. 10 patients with PPCS, and 9 patients without, had missing vocabulary scores.

1 *p* value from mTBI/Controls comparison.

2 *p* value from PPCS+/PPCS- comparison. No statistical comparisons were performed for cause of injury, GCS, and level of care because of low *n* in some cells.

3 Mann-Whitney U-test.

4 Chi-squared test.

5 t-test.

6 Multiple regression with age and sex as covariates.

mTBI, mild traumatic brain injury; PPCS+/PPCS-, patients with and without persistent post-concussion symptoms; IQR, interquartile range; SD, standard deviation; GCS, Glasgow Coma Scale; LOC, loss of consciousness; PTA, post-traumatic amnesia.

A total of 35 patients (20%) with mTBI met the criteria for PPCS at 3 months. Longer post-traumatic amnesia (PTA) and complicated mTBI were more common in patients with PPCS than in patients without PPCS. Further, patients with PPCS were more often women and had lower vocabulary scores (Table 1).

### TBSS: Patients with PPCS versus without PPCS

**FIG. 1.**
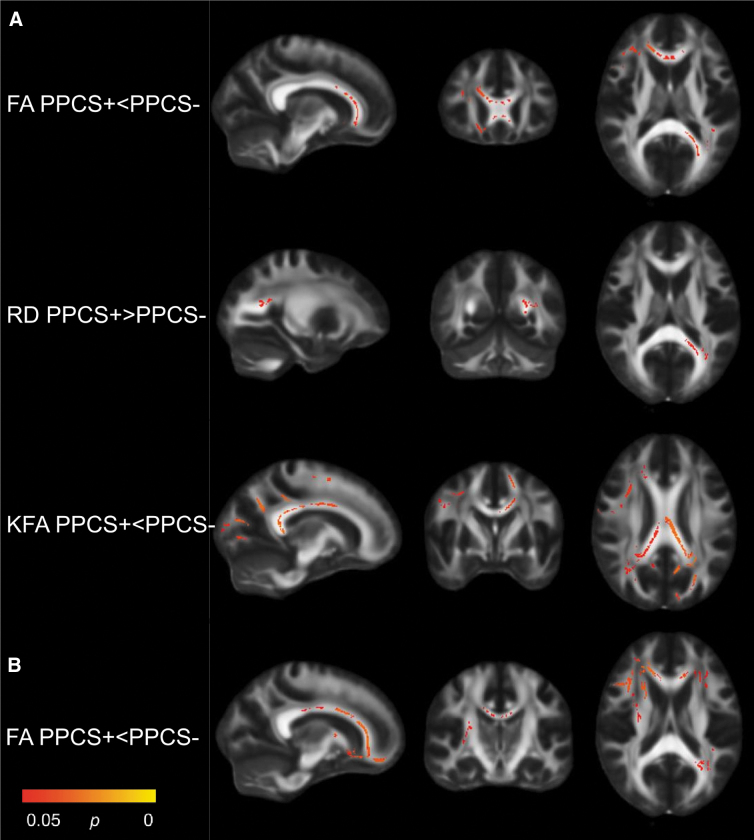
Significant contrasts from Tract-Based Spatial Statistics: PPCS+ vs PPCS-. Patients with (PPCS+) vs without (PPCS-) persistent post-concussion symptoms; total sample **(A),** uncomplicated sample **(B).** Statistically significant voxels in red and yellow. FA, fractional anisotropy; KFA, kurtosis fractional anisotropy; RD, radial diffusivity.

In the total sample, patients with PPCS had significantly lower FA (in 2.7% of all voxels analyzed) and KFA (in 6.9% of all voxels) and higher RD (in 0.3% of all voxels) than patients without PPCS (Table 2). The differences were mainly located in the corpus callosum, corona radiata, internal capsule, and thalamic radiation (Fig. 1A). In the uncomplicated sample (PPCS + *n* = 27; PPCS- *n* = 1 31), patients with PPCS had lower FA than patients without PPCS in a larger number of voxels (5.0%) and more widespread than in the total sample, but no significant differences in KFA or RD were present (Table 2; Fig. 1B).

**Table 2.** Comparisons with Significant Findings: Number of Participants, Direction of Change, Lowest Adjusted *p* Value, and the Number and Percentage of Significant Voxels

Comparison	*n*	Direction < / >	Lowest* p *value	Number of significant voxels	% of total voxels
PPCS+ vs. PPCS-					
Total sample					
FA	35/141	PPCS+<PPCS-	0.035	3695	2.7
RD	35/141	PPCS+>PPCS-	0.044	411	0.3
KFA	35/141	PPCS+<PPCS-	0.026	9544	6.9
Uncomplicated sample					
FA	27/131	PPCS+<PPCS-	0.028	6950	5.0
PPCS+ vs. Controls					
Total sample					
FA	35/78	PPCS+<Controls	0.002	12818	9.3
MD	35/78	PPCS+>Controls	0.019	25329	18.4
AD	35/78	PPCS+>Controls	0.015	2245	1.6
RD	35/78	PPCS+>Controls	0.005	24590	17.8
KFA	35/78	PPCS+<Controls	0.006	25387	18.4
Kmean	35/78	PPCS+<Controls	0.032	7600	5.5
Kax	35/78	PPCS+<Controls	0.028	5244	3.8
Krad	35/78	PPCS+<Controls	0.026	9780	7.1
Uncomplicated sample					
FA	27/78	PPCS+<Controls	0.003	22422	16.3
MD	27/78	PPCS+>Controls	0.028	15915	11.5
RD	27/78	PPCS+>Controls	0.010	26144	19.0
KFA	27/78	PPCS+<Controls	0.018	20456	14.8
Kmean	27/78	PPCS+<Controls	0.027	16713	12.1
Kax	27/78	PPCS+<Controls	0.050	3	< 0.1
Krad	27/78	PPCS+<Controls	0.027	19553	14.2
Adjusted for vocabulary					
MD, Total sample	25/74	PPCS+>Controls	0.038	6743	4.9
AD, Total sample	25/74	PPCS+>Controls	0.029	1059	0.8
RD, Total sample	25/74	PPCS+>Controls	0.047	292	0.2
RD, Uncomplicated	19/74	PPCS+>Controls	0.049	212	0.2
PPCS- vs. Controls					
Total sample					
Kmean	141/78	PPCS-<Controls	0.029	7092	5.1
Kax	141/78	PPCS-<Controls	0.035	333	0.2
Krad	141/78	PPCS-<Controls	0.036	4970	3.6
Uncomplicated sample					
Kmean	131/78	PPCS-<Controls	0.040	3978	2.9
Krad	131/78	PPCS-<Controls	0.030	6533	4.7
Adjusted for vocabulary					
Kmean, Total sample	132/74	PPCS-<Controls	0.023	11258	8.2
Kax, Total sample	132/74	PPCS-<Controls	0.025	825	0.6
Krad, Total sample	132/74	PPCS-<Controls	0.036	4838	3.5
Kmean, Uncomplicated	123/74	PPCS-<Controls	0.026	10318	7.5
Krad, Uncomplicated	123/74	PPCS-<Controls	0.030	9455	6.9

The table shows the *p* value for the voxel with the lowest *p* value, the direction, and the number of significant voxels. For example, for FA, when comparing patients with PPCS (*n* = 35) to patients without PPCS (*n* = 141) in the total sample, patients with PPCS had significantly *lower* FA in 3695 voxels, which equals 2.7% of the total number of voxels (137,832). The lowest adjusted *p* value among these voxels was 0.035.

PPCS+/-, patients with and without persistent post-concussion symptoms; FA, fractional anisotropy; RD, radial diffusivity; KFA, kurtosis fractional anisotropy; Kmean, kurtosis mean; Kax, axial kurtosis; Krad, radial kurtosis; MD, mean diffusivity; AD, axial diffusivity.

When vocabulary was included as a covariate, no significant differences remained between patients with and without PPCS neither in the total sample nor in the uncomplicated sample.

### TBSS: Patients with PPCS versus controls

**FIG. 2.**
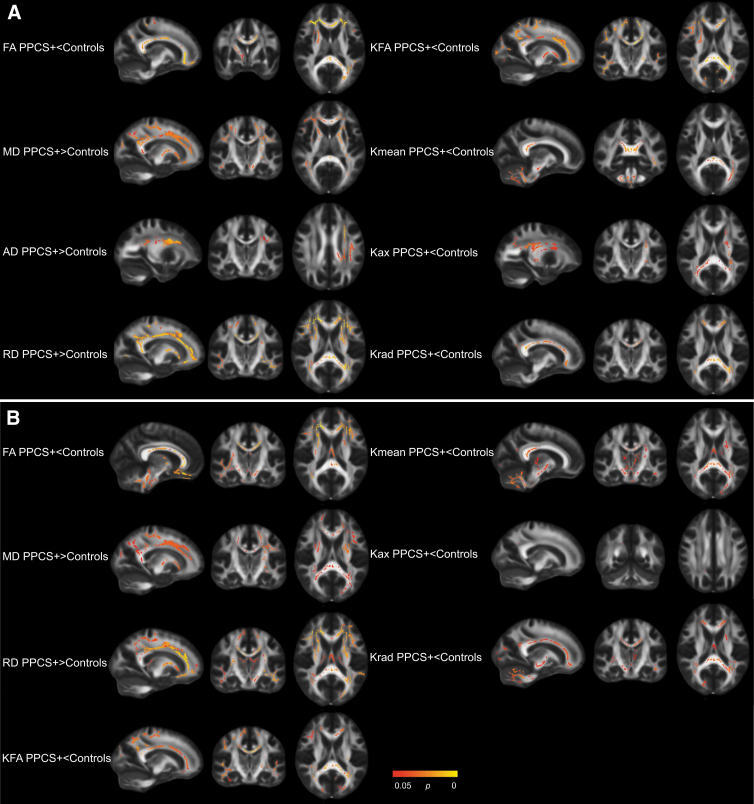
Significant contrasts from Tract-Based Spatial Statistics: PPCS+ vs Controls. Patients with persistent post-concussion symptoms (PPCS+) versus healthy controls; total sample **(A),** uncomplicated sample **(B).** Statistically significant voxels in red and yellow. AD, axial diffusivity; FA, fractional anisotropy; Kax, axial kurtosis; KFA, kurtosis fractional anisotropy; Kmean, kurtosis mean; Krad, radial kurtosis; MD, mean diffusivity; RD, radial diffusivity.

In the total sample, patients with PPCS differed from controls on all diffusion metrics examined (i.e., patients with PPCS had lower FA, KFA, Kmean, Kax, and Krad, and higher MD, AD, and RD; Table 2). The differences were widespread, including voxels in the corpus callosum, corona radiata, internal capsule, superior longitudinal fasciculus, and thalamic radiation. In particularly for Kmean, group differences also were present in the cerebellum and brainstem (Fig. 2A). In the uncomplicated sample, significant differences in all metrics but AD remained. As in the total sample, the differences were widespread (Table 2; Fig. 2B).

When vocabulary was included as a covariate in the total sample, significant differences between patients with PPCS and controls in MD, AD, and RD remained, but the number of significant voxels was reduced (Table 2). When vocabulary was included as a covariate in the uncomplicated sample, only differences in RD (in <0.1% of all voxels) remained significant (Table 2).

### TBSS: Patients without PPCS versus controls

**FIG. 3.**
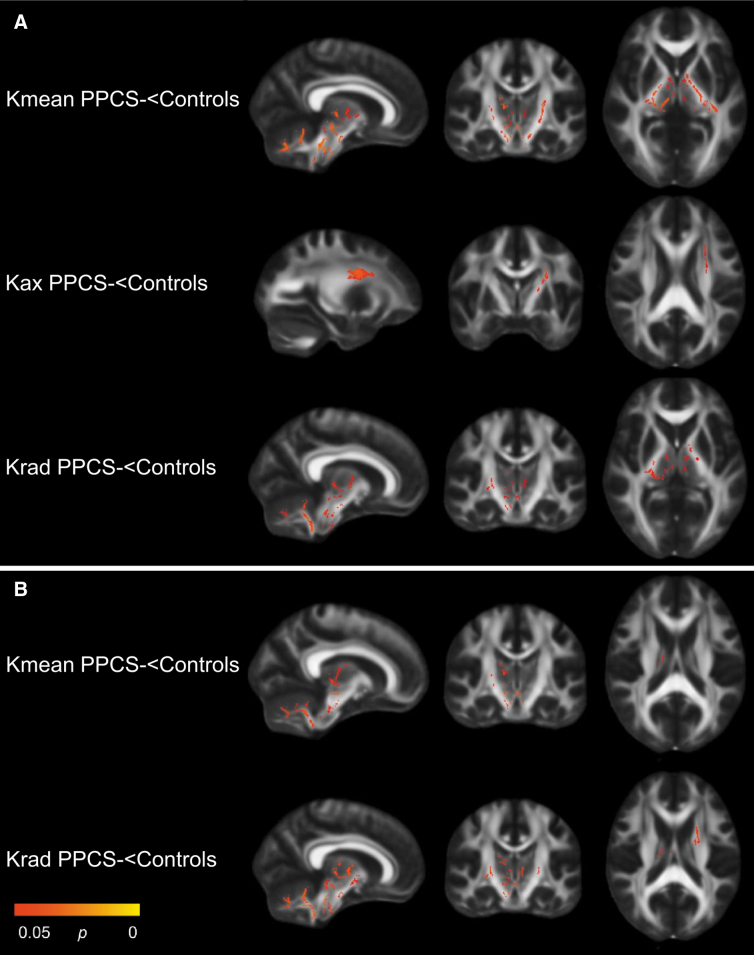
Significant contrasts from Tract-Based Spatial Statistics: PPCS- vs Controls. Patients without persistent post-concussion symptoms (PPCS-) versus healthy controls, total sample **(A),** uncomplicated sample **(B).** Statistically significant voxels in red and yellow. Kax, axial kurtosis; Kmean, kurtosis mean; Krad, radial kurtosis.

In the total sample, patients without PPCS had lower kurtosis metrics (Kmean, Kax, and Krad) than controls. The findings were mainly located in the internal capsule, cerebellum, brainstem, and thalamus (Table 2; Fig. 3A). In the uncomplicated sample, patients without PPCS had lower Kmean and Krad (Table 2; Fig. 3B). Results from the comparisons between patients without PPCS and controls were generally unaffected by including vocabulary as a covariate (Table 2).

### TBSS: Vocabulary and diffusion metrics

Due to the weakened associations between PPCS and diffusion metrics when vocabulary was included as a covariate, we performed a series of follow-up analyses. However, we found no significant associations between vocabulary scores and diffusion metrics in the total sample, in patients with mTBI only, or in controls only. Further, we found no significant interaction effects (group*vocabulary) that would have indicated that the association between vocabulary and diffusion metrics differed between patients with mTBI and healthy controls.

## Discussion

In this large prospective study on patients with mTBI who underwent MRI in the acute phase, we found that patients who later developed PPCS had poorer white matter microstructural integrity than patients who recovered. Differences also were present when patients with complicated mTBI were excluded from analyses. However, when we controlled for cognitive reserve, the differences in white matter integrity were reduced, which suggests that the observed differences in diffusion metrics were not exclusively caused by the mTBI, but also by pre-existing deviations in white matter microstructure in patients with PPCS.

Patients who developed PPCS had poorer white matter integrity than patients who did not develop PPCS, as indicated by lower FA and KFA, and higher RD. When we excluded patients with complicated mTBI, only differences in FA remained, suggesting that reduction in FA was a robust finding. To our knowledge, the current study is the first large study on mixed-mechanism mTBI demonstrating low FA in the acute phase in patients who later develop PPCS. In our pilot study, we found no differences in DTI or DKI metrics assessed within 72 h, between patients who had or did not have PPCS 3 months later. However, the sample included just nine patients with PPCS, increasing the uncertainty of the results.^32^

The finding of acutely lower FA extends previous findings from civilian mixed-mechanism studies (sample size range 16 to 134), reporting associations between PPCS and lower FA in the subacute (i.e., around or after 2 weeks)^5,6,8^ or chronic (i.e., after 3 months) phase.^9–11^ In the present study, differences between patients with PPCS and without PPCS were mainly located in the corpus callosum, corona radiata, internal capsule, and thalamic radiation. In previous subacute and chronic studies, it varies in which white matter tracts differences are observed, but differences in these tracts, especially in the corpus callosum, are commonly reported.^5,6,9,10,12,52^ In contrast to studies on mixed-mechanism mTBI, studies on sports-related concussion have acquired acute MRI more frequently, and a few studies have reported associations between higher MD, AD, and RD and symptoms,^53,54^ but a lack of associations also has been reported.^35,55^ However, the symptoms also were assessed acutely in most studies on sports-related concussion, which makes it difficult to directly compare these findings with ours. Moreover, the variability in both age and injury mechanism is considerably larger in mixed-mechanism studies.

When we compared patients with PPCS and controls, profound differences were found in white matter integrity, indicated by widespread and significant differences in all eight DTI and DKI metrics examined. That differences in diffusion metrics between patients with PPCS and controls were greater than between patients with and without PPCS, have previously been reported in the subacute phase,^7,56^ and the present results extend these findings to the acute phase. Notably, in the present study, the differences in nearly all metrics remained when patients with complicated mTBI were excluded. Consequently, acute DTI and DKI have value in PPCS prediction above that of the clinical MRI, extending previous observations of the added value of subacute DTI in outcome prediction.^8^ However, it should be noted that the use of DTI and DKI in clinical practice is in its infancy. The methods are presently resource-demanding, no norm data are implemented in clinical analysis software, and scanner and scan acquisition protocol effects complicate the clinical implementation further. Moreover, future research should examine the added predictive value of DTI and DKI measures in models comprising also other established risk factors for PPCS.

When patients without PPCS were compared with controls, differences were only observed in DKI metrics. This finding shows that DKI may be more sensitive than DTI to mTBI -induced alteration in white matter, and that mTBI seems to induce alterations that are not associated with PPCS. These findings are in line with a few previous studies reporting that DKI was superior to DTI in detecting deviations in white matter in the acute to chronic phase after mTBI.^33,35^ The present results add to this literature by showing that although DKI metrics were affected acutely by the mTBI, the DTI metrics were more closely related to PPCS development.

The general finding from the present study is that mTBI is associated with lower FA, higher MD (and the MD subcomponents AD and RD), and lower kurtosis values, shortly after the injury. While this direction of change aligns with most previous research,^3^ opposite patterns such as higher FA^57^ and kurtosis values^34,35^ also have been reported in the acute phase. These inconsistences may be related to sample differences in injury type, severity, and the time-point of the MRI. Higher FA and lower MD after mTBI have been interpreted as representing cytotoxic edema, which may be more severe than the vasogenic edema suggested to underlie decreased FA and increased MD.^4,58,59^ In the present sample, however, we found no evidence for increased FA in the acute phase.

Cognitive reserve appeared to contribute to the observed differences in diffusion metrics. None of the diffusion metrics remained significantly different between patients with and without PPCS when cognitive reserve was adjusted for. A common finding in the mTBI literature is that patients with PPCS, on average, differ from patients without PPCS on several pre-injury factors, such as having poorer pre-injury mental and physical health^60^ and lower educational attainment^61^ and estimated intelligence.^24^ However, this is rarely accounted for in DTI and DKI studies on mTBI, despite that some of these pre-injury factors have been associated with deviations in DTI and DKI metrics outside the mTBI research context. Indeed, lower intelligence,^19,20^ headache,^17^ and depression^16^ have been associated with poorer white matter microstructural integrity. Thus, even though deviations in diffusion metrics after mTBI have been demonstrated in animal experimental designs,^4^ abnormal diffusion is not specific to mTBI.

It is possible that the mixed findings in DTI and DKI studies on PPCS result from pre-injury differences between those prone to develop PPCS and those who do not. Such differences may vary between studies, due to variations in participant recruitment. Our findings can be understood within a reserve framework, where high cognitive and brain (e.g., white matter integrity) reserve protects against aversive effects of brain trauma.^25^ Nonetheless, when patients with PPCS were compared with the control group in the present study, several differences in diffusion metrics were significant, even when cognitive reserve was controlled for. Further, comparisons between patients without PPCS and controls were generally unaffected by including cognitive reserve as a covariate.

Also, in separate, follow-up analyses, we did not find any clusters of voxels with statistically significant associations between the proxy of cognitive reserve, vocabulary scores, and diffusion metrics, suggesting that this association is weak. Thus, although pre-existing differences in cognitive reserve seemed to explain some of the deviations in white matter microstructure observed in patients with PPCS, the mTBI also appeared to contribute to differences in diffusion metrics. If patients at risk of PPCS differ in pre-injury axonal integrity, this is probably associated with a range of factors (e.g., poorer physical and mental health), and cognitive reserve might be just one of several contributing factors.

### Limitations

Although this study addressed some shortcomings of previous studies, by having a larger sample and an early uniform time-point of MRI, the study also has limitations. First, despite a large number of patients, different findings in the total sample and the uncomplicated mTBI sample could partly be caused by the reduction of statistical power when cases were excluded. Similarly, 23 (9%) of the participants were not assessed with the Vocabulary subtest (number of cases in each contrast shown in Table 2). Second, cognitive reserve and intelligence were estimated from the result on a single test, increasing the uncertainty of this measure. Intelligence is preferably measured with a battery of tests, such as the Wechsler Adult Intelligence Scale. However, in studies on brain injury, it is important that results on tests of intelligence are not affected by the injury (i.e., that they can estimate pre-injury functioning). Therefore, single tests known to be largely insensitive to brain pathology are usually used. These tests are often language-based and measure vocabulary knowledge or word reading in different varieties.^45,62^ Third, other indices of pre-existing vulnerability might be more valid and relevant than cognitive reserve estimated by intelligence, and should be explored in future studies. Finally, this study analyzed the diffusion data voxel-wise, with threshold-free cluster enhancement. One limitation of this method is that mediation analyses are difficult to implement. Therefore, it is uncertain to what extent the association between PPCS and diffusion metrics was weakened by cognitive reserve. Future studies need to investigate this further, with different methods.

## Conclusion

In this large study on mTBI, patients who later developed PPCS had poorer white matter microstructural integrity in the acute phase than patients without PPCS and healthy controls. This finding also was evident in patients with uncomplicated mTBI. However, some of these differences in diffusion metrics could be ascribed to pre-existing differences between the groups and the individual vulnerability of developing PPCS might therefore be related to pre-injury variability in axonal integrity. In summary, we suggest that certain white matter characteristics could represent both a pre-injury risk factor and an injury-related biomarker for poor outcome after mTBI. The current study shows that careful consideration of pre-injury differences including but not restricted to typical proxies of cognitive reserve is warranted in diffusion MRI studies when comparing patients with good and poor outcome.

## Supplementary Material

Supplemental data
