# Supplementary material for: Acute Diffusion Tensor and Kurtosis Imaging and Outcome following Mild Traumatic Brain Injury
Source: J Neurotrauma. 2021 Aug 23;38(18):2560–71. doi: 10.1089/neu.2021.0074 (PMC8403189; doi:10.1089/neu.2021.0074)
Supplement: Supplemental data [file Supp_Data.docx]

**Supplementary Online Content**

Stenberg J, Eikenes L, Moen KG, Vik A, Håberg AK, Skandsen T. Acute Diffusion Tensor and Kurtosis Imaging and Outcome Following Mild Traumatic Brain Injury.

**Methods and Materials**

***Inclusion and Exclusion Criteria***

The TBI was defined as mild per the WHO Collaborating Centre Task Force on Mild Traumatic Brain Injury criteria: 1) Glasgow Coma Scale score (GCS) 13-15 at presentation in the emergency room; 2) loss of consciousness (LOC) < 30 minutes; and (3) post-traumatic amnesia (PTA) < 24 hours.^1^ PTA was defined as the self-reported time after injury for which the patient had no memory, and LOC had to be witnessed in order to be defined as present. Exclusion criteria were (1) non-fluency in the Norwegian language; (2) pre-existing severe neurological disorder (e.g., stroke, multiple sclerosis), or a prior history of a complicated mild, moderate, or severe TBI; or (3) ongoing severe psychiatric (e.g., psychotic or bipolar disorder), somatic (e.g., cancer), or substance abuse problems, determined by the researcher responsible for inclusion and considered to be severe enough to likely interfere with follow-up. The exclusion criteria applied in the MTBI group were used for the controls, but in addition, controls receiving treatment for severe psychiatric disorders were excluded, even if they might be able to comply with follow-up. The latter criterion was slightly different for patients and controls, because the goal was to establish an MTBI cohort as representative as possible, and a control group with reasonably good brain health.

***DTI and DKI Data Processing***

The DTI/DKI sequence was a single-shot balanced‐echo EPI sequence acquired in 30 non‐collinear directions with 3 b‐values (b = 0, b = 1,000 and, b = 2,000 s/mm2). The following parameters were used: TR 8,800 ms, TE 95 ms, FOV 240 × 240 mm, slice thickness 2.5 mm, acquisition matrix 96 × 96. Sixty transversal slices with no gaps were acquired, giving full brain coverage. Five images without diffusion weighting were acquired to increase signal‐to‐noise ratio. To correct for image distortion, two additional b0 images were acquired with opposite phase encoding polarity.^2^

Image analyses were performed with the fMRIB Software Library (FSL: http://www.fmrib.ox.ac.uk/fsl) and the Diffusion Kurtosis Estimator (DKE: https://medicine.musc.edu/departments/centers/cbi/dki/dki-data-processing). Non‐brain tissue was removed with the Brain Extraction Tool (FSL). Artifacts due to eddy currents and movements were corrected with eddy (FSL). Correction of the susceptibility‐induced off‐resonance field artifacts was done by topup (FSL). DKI and DTI model fitting was performed using DKE and parametric maps and were calculated for 8 metrics: Fractional Anisotropy (FA), mean diffusivity (MD), axial diffusivity (AD), radial diffusivity (RD), kurtosis fractional anisotropy (KFA), mean kurtosis (Kmean), axial kurtosis (Kax), and radial kurtosis (Krad).^3^

FA represents the directional restriction of movement (i.e., the amount of anisotropic diffusion, and not the magnitude of diffusion). MD represents the mean diffusion in all directions. AD represents the diffusion along the direction of primary movement (e.g., along the axon in a healthy brain). RD represents the mean movement in the other two directions (i.e., excluding the direction of the primary movement).^4^ In previous studies, findings on the direction of change in acute DTI metrics after MTBI are inconsistent, but many argue that lower FA and higher MD is indicative of poorer white matter integrity, and possibly axonal injury.^5–8^ As with FA, KFA measure the anisotropy of diffusion.^9^ Kmean, Kax, and Krad measure the kurtosis (i.e., the deviation from a Gaussian distribution) in different diffusion directions. Thus, values closer to zero indicate a diffusion of water molecules that is less restricted, approaching a Gaussian distribution,^10^ which may be indicative of reduced tissue heterogeneity, and possibly, neuronal damage.^11^

Voxel-wise statistical analysis of the diffusion data was performed using Tract-Based Spatial Statistics (TBSS).^12^ Briefly, all subjects' FA data were aligned into a common space using the non-linear registration tool FNIRT^13,14^ (which uses a b-spline representation of the registration warp field).^15^ A mean FA image was created from all the FA images and thinned to create a skeletonized mean FA representing the centers of all tracts common to all the subjects in the analysis. The mean FA skeleton was thresholded to FA ≥ 0.2 to include major white matter tracts, but exclude peripheral tracts and grey matter. Each subject's aligned FA data were then projected onto this skeleton. The skeletonization process was also applied to MD, AD, RD, KFA, Kmean, Kax and Krad, and the statistical comparisons of these data were then restricted to voxels in the white matter skeleton. The resulting skeletonized data were consequently fed into voxel-wise cross-subject statistics in Randomise.

**Supplementary Table 1.** Injury- and demographic characteristics of patients in the extended follow-up (total n=199) included and not included in the present study.

|  | **Included** | **Not Included** | ***p*** |
| --- | --- | --- | --- |
| *n* | 176 | 23 |  |
| Age, years, median (IQR) | 28.1 (22.0) | 19.7 (11.9) | .001^1^ |
| Female sex, *n* (%) | 65 (36.9) | 8 (34.8) | .841^2^ |
| Education, years, median (IQR) | 13.0 (4.0) | 12.0 (2.0) | .002^1^ |
| Vocabulary |  |  |  |
| T score, mean (SD) | 50.9 (9.2) | 52.2 (8.5) | .662^3^ |
| Raw score, mean (SD) | 57.4 (8.6) | 56.9 (8.2) | .893^4^ |
| Cause of injury, *n* (%) |  |  |  |
| Fall | 68 (38.6) | 9 (39.1) |  |
| Bicycle | 33 (18.8) | 2 (8.7) |  |
| Violence | 23 (13.1) | 5 (21.7) |  |
| Sports accidents | 21 (11.9) | 5 (21.7) |  |
| Motor vehicle accidents | 17 (9.7) | 2 (8.7) |  |
| Hit by object | 12 (6.8) | 0 (0) |  |
| Other | 1 (0.6) | 0 (0) |  |
| Unknown | 1 (0.6) | 0 (0) |  |
| GCS score, *n* (%) |  |  |  |
| 13 | 4 (2.3) | 1 (4.3) |  |
| 14 | 25 (14.2) | 7 (30.4) |  |
| 15 | 136 (77.3) | 15 (65.2) |  |
| unknown | 11 (6.3) | 0 (0) |  |
| LOC, *n* (%) |  |  |  |
| Yes | 85 (48.3) | 10 (43.5) |  |
| No | 30 (17.0) | 4 (17.4) |  |
| unknown/not witnessed | 61 (34.7) | 9 (39.1) |  |
| PTA, *n* (%) |  |  | .380^2^ |
| < 1 hour | 123 (69.9) | 14 (60.9) |  |
| 1-24 hours | 53 (30.1) | 9 (39.1) |  |
| Complicated MTBI, *n* (%) |  |  | .021^2^ |
| Yes | 18 (10.2) | 6 (27.3) |  |
| No | 158 (89.8) | 16 (72.7) |  |
| Level of Care, *n* (%) |  |  |  |
| Not admitted | 124 (70.5) | 12 (52.2) |  |
| Observed < 24 hours | 27 (15.3) | 4 (17.4) |  |
| Admitted neurosurgery department | 16 (9.1) | 7 (30.4) |  |
| Admitted other department | 9 (5.1) | 0 (0) |  |

Note: No statistical comparisons were performed for cause of injury, GCS, LOC, and level of care because of low *n* in some cells; ^1^Mann-Whitney U-test; ^2^Chi-square test; ^3^t-test; ^4^Multiple regression with age and sex as covariates. GCS = Glasgow Coma Scale; LOC = Loss of Consciousness; MTBI= Mild Traumatic Brain Injury; PTA = Post-Traumatic Amnesia

**References**

1. Carroll, L.J., Cassidy, J.D., Holm, L., Kraus, J., and Coronado, V.G. (2004). Methodological issues and research recommendations for mild traumatic brain injury: the WHO collaborating centre task force on mild traumatic brain injury. J. Rehabil. Med. 43, 113–125.

2. Holland, D., Kuperman, J.M., and Dale, A.M. (2010). Efficient correction of inhomogeneous static magnetic field-induced distortion in Echo Planar Imaging. Neuroimage 50, 175–183.

3. Tabesh, A., Jensen, J.H., Ardekani, B.A., and Helpern, J.A. (2011). Estimation of tensors and tensor-derived measures in diffusional kurtosis imaging. Magn. Reson. Med. 65, 823–36.

4. Soares, J.M., Marques, P., Alves, V., and Sousa, N. (2013). A hitchhiker’s guide to diffusion tensor imaging. Front. Neurosci. 7, 31.

5. Khong, E., Odenwald, N., Hashim, E., and Cusimano, M.D. (2016). Diffusion tensor imaging findings in post-concussion syndrome patients after mild traumatic brain injury: a systematic review. Front Neurol 7, 156.

6. Wallace, E.J., Mathias, J.L., and Ward, L. (2018). Diffusion tensor imaging changes following mild, moderate and severe adult traumatic brain injury: a meta-analysis. Brain Imaging Behav. 12, 1607–1621.

7. Niogi, S.N., and Mukherjee, P. (2010). Diffusion tensor imaging of mild traumatic brain injury. J. Head Trauma Rehabil. 25, 241–255.

8. Hutchinson, E.B., Schwerin, S.C., Avram, A. V., Juliano, S.L., and Pierpaoli, C. (2018). Diffusion MRI and the detection of alterations following traumatic brain injury. J. Neurosci. Res. 96, 612–625.

9. Glenn, G.R., Helpern, J.A., Tabesh, A., and Jensen, J.H. (2015). Quantitative assessment of diffusional kurtosis anisotropy. NMR Biomed. 28, 448–59.

10. Steven, A.J., Zhuo, J., and Melhem, E.R. (2014). Diffusion kurtosis imaging: an emerging technique for evaluating the microstructural environment of the brain. AJR. Am. J. Roentgenol. 202, W26-33.

11. Grossman, E.J., Ge, Y., Jensen, J.H., Babb, J.S., Miles, L., Reaume, J., Silver, J.M., Grossman, R.I., and Inglese, M. (2012). Thalamus and cognitive impairment in mild traumatic brain injury: a diffusional kurtosis imaging study. J. Neurotrauma 29, 2318–2327.

12. Smith, S.M., Jenkinson, M., Johansen-Berg, H., Rueckert, D., Nichols, T.E., Mackay, C.E., Watkins, K.E., Ciccarelli, O., Cader, M.Z., Matthews, P.M., and Behrens, T.E.J. (2006). Tract-based spatial statistics: voxelwise analysis of multi-subject diffusion data. Neuroimage 31, 1487–1505.

13. Anderson, J., Jenkinson, M., and Smith, S. (2007). Non-linear registration, aka spatial normalisation. FMRIB technical report TR07JA2.

14. Anderson, J., Jenkinson, M., and Smith, S. (2007). Non-linear registration optimisation. FMRIB technical report TR07JA1.

15. Rueckert, D., Sonoda, L., Hayes, C., Hill, D., Leach, M., and Hawkes, D. (1999). Nonrigid registration using free-form deformations: application to breast MR images. IEEE Trans. Med. Imaging 18, 712–721.
